# Refractory Antiphospholipid Syndrome With Thoracic Outlet and Superior Vena Cava Syndromes: A Complex Case of Recurrent Thrombosis

**DOI:** 10.7759/cureus.94557

**Published:** 2025-10-14

**Authors:** Talha A Khan, Sam Schick, Jacqueline Figueroa, Jay Swami

**Affiliations:** 1 Surgery, Ross University School of Medicine, Weston, USA; 2 Surgery, Cleveland Clinic Weston Hospital, Weston, USA; 3 Interventional Radiology, American University of the Caribbean School of Medicine, Cupecoy, SXM; 4 Internal Medicine, Ross University School of Medicine, Weston, USA

**Keywords:** antiphospholipid syndrome, hereditary angioedema, mast cell activation, superior vena cava syndrome, thoracic outlet syndrome

## Abstract

Thoracic outlet syndrome (TOS) and superior vena cava syndrome (SVCS) are rare and debilitating conditions that can cause vascular compression, hypercoagulable states, and central venous obstruction. This is the case of a 39-year-old female with treatment-resistant antiphospholipid syndrome (APS), hereditary angioedema (HAE), and mast cell activation syndrome (MCAS) complicated by SVCS and venous TOS. Her clinical course was further challenged by recurrent venous thromboembolism with treatment failure on multiple direct oral anticoagulants (DOACs), with warfarin being the only effective medication. Furthermore, the patient experienced several thrombotic events, which included: left subclavian vein thrombosis, recurrent deep vein thromboses (DVTs), and septic thrombophlebitis (methicillin-sensitive *Staphylococcus epidermidis *bacteremia) involving the left internal jugular vein. Her vascular history also included left lower lobectomy for pulmonary nodules and right cerebral stenting for reversible cerebral vasoconstriction syndrome (RCVS). The patient also had gastroparesis attributed to HAE-related visceral edema. This case illustrates the challenges posed by overlapping rare disorders, where the combination of APS and mechanical venous obstruction resulted in recurrent thromboses, complicated further by the patient’s complex past medical history. Our findings highlight the limitations of conventional anticoagulation strategies in treatment-resistant APS and emphasize the need for multidisciplinary approaches.

## Introduction

Thoracic outlet syndrome (TOS) and superior vena cava syndrome (SVCS) are disorders that result from the compression of central venous structures. Venous TOS, a subtype accounting for only 3%-5% of all TOS cases, typically presents with upper extremity swelling, pain, and venous distention due to compression of the subclavian vein between the clavicle and first rib [1,2]. SVCS, associated with malignancy or long-term intravascular devices, manifests with impaired venous return from the head, neck, and upper extremities, leading to facial edema, dyspnea, and prominent thoracic collaterals [3,4]. Individually, these conditions are rare, but in this patient, the combination of these conditions poses a significant challenge for the treatment protocol.

Given the complex nature of this presentation, it is further confounded when combined with antiphospholipid syndrome (APS), as was the case. APS is described as an autoimmune condition characterized by the presence of antiphospholipid antibodies in association with recurrent arterial or venous thromboembolic events [5]. Standard anticoagulation with vitamin K antagonists such as warfarin is considered the gold standard treatment, particularly in high-risk patients with triple antibody positivity. However, even when patients are taking warfarin correctly, thromboembolic events can occur. Direct oral anticoagulants have demonstrated inferior efficacy in this population [6,7]. A meta-analysis conducted on anticoagulation failure in APS demonstrated a need for individualized management strategies, which included warfarin and perioperative bridging with parenteral agents [8]. Complement inhibitors such as eculizumab have been used as salvage therapy, particularly in APS or recurrent thrombotic disease unresponsive to standard regimens, with case reports and small series suggesting superiority.

The case is further challenged in patients with concurrent hereditary angioedema (HAE) and mast cell activation syndrome. HAE, a rare disorder caused by deficiency or dysfunction of C1 esterase inhibitors, can cause visceral edema and gastrointestinal dysmotility [9]. This often leads to enteral feeding intolerance and prolonged reliance on central venous access for nutrition, further increasing the risk for thromboembolic events [10]. Mast cell activation may also contribute to vascular instability and medication hypersensitivity, which further demonstrates the need for individualized planning strategies among multiple disciplines [11].

Emerging evidence also highlights the potential role of X chromosome inactivation (XCI) abnormalities in predisposing women to immune dysregulation. Incomplete or skewed XCI can lead to the overexpression of X-linked immune-related genes, such as TLR7, CD40L, and IRAK1, thereby enhancing innate and adaptive immune responses [12]. This mechanism may underlie the observed female predominance in autoimmune diseases such as APS and mast cell disorders. While not directly causative, such X-linked immune amplification may contribute to the prothrombotic and inflammatory milieu seen in this case, particularly in the setting of overlapping immune and vascular syndromes.

This case report describes a 39-year-old woman with treatment-refractory APS, HAE, mast cell activation, and bilateral venous compression syndromes. Despite high-intensity warfarin, she developed multiple thrombotic events, including but not limited to septic thrombophlebitis. The patient also experienced similar events in previously stented areas. The hospital course for this patient required the recruitment of multiple departments, including vascular and thoracic surgery, hematology, gastroenterology, and immunology. This case illustrates the clinical consequences of intersecting autoimmune, vascular, and immunologic pathology and highlights the limitations of current anticoagulation strategies.

## Case presentation

A 39-year-old woman with a complex medical history presented to the emergency department on June 11, 2025, with five days of right upper extremity pain, fever (maximum temperature of 102.8°F), neck and shoulder pain, and chills. Her past medical history included mast cell activation syndrome, hereditary angioedema, dysautonomia with inappropriate sinus tachycardia, iron deficiency anemia, gastroparesis requiring chronic total parenteral nutrition (TPN), and SVCS treated with multiple venous stents, and treatment-resistant APS (diagnosed January 2016) confirmed by persistently positive IgG anticardiolipin antibodies and lupus anticoagulant meeting the International Society of Thrombosis and Hemostasis (ISTH) criteria, even in the setting of supra-therapeutic warfarin levels, as demonstrated in laboratory testing from December 2021. These findings indicated high-risk APS, requiring lifelong high-intensity anticoagulation. Her vascular history included a left lower lobectomy in 2020 for pulmonary nodules and right cerebral stenting in 2019 for reversible cerebral vasoconstriction syndrome. She had multiple venous stents in the left brachiocephalic artery, subclavian, and axillary veins placed for SVCS and venous TOS.

Her thrombotic history was notable for left subclavian vein thrombosis in November 2023, recurrent deep vein thrombosis (DVT) in February 2025, and septic thrombophlebitis of the left internal jugular vein with methicillin-sensitive *Staphylococcus epidermidis *(MSSE) bacteremia in April 2025. She had failed multiple direct oral anticoagulants that included apixaban, rivaroxaban, and fondaparinux. The patient also experienced bleeding complications on Lovenox. Since 2018, she has been maintained on high-intensity Coumadin warfarin targeting an INR of 4-5.

On the initial presentation, the patient's INR was 6.0. Duplex ultrasound demonstrated an acute right upper extremity DVT, indicating possible treatment failure with Coumadin. Blood cultures grew MSSE, consistent with recurrent line-associated infection. The patient had recently completed intravenous cefazolin for methicillin-sensitive Staphylococcus aureus bacteremia related to a prior internal jugular DVT. Transesophageal echocardiography and PET/CT showed no evidence of endocarditis or occult infection. The left upper extremity port was removed, and a right groin central venous catheter was placed for TPN. Following removal, repeat blood cultures were negative, and cefazolin was discontinued on June 18.

On hospital day five, she developed a new left upper extremity DVT. Warfarin was held, and intravenous unfractionated heparin was initiated pending surgical intervention. CT angiography with venous phase showed mild in-stent stenosis with preserved flow.

Nutritional management was complicated by gastroparesis and mast cell activation syndrome. Endoscopic placement of a Corpak feeding tube on June 24 failed due to intragastric looping, and the tube was removed on June 30. She remained dependent on TPN via her temporary central venous catheter, with plans for port reinsertion following infectious clearance.

**Figure 1:**
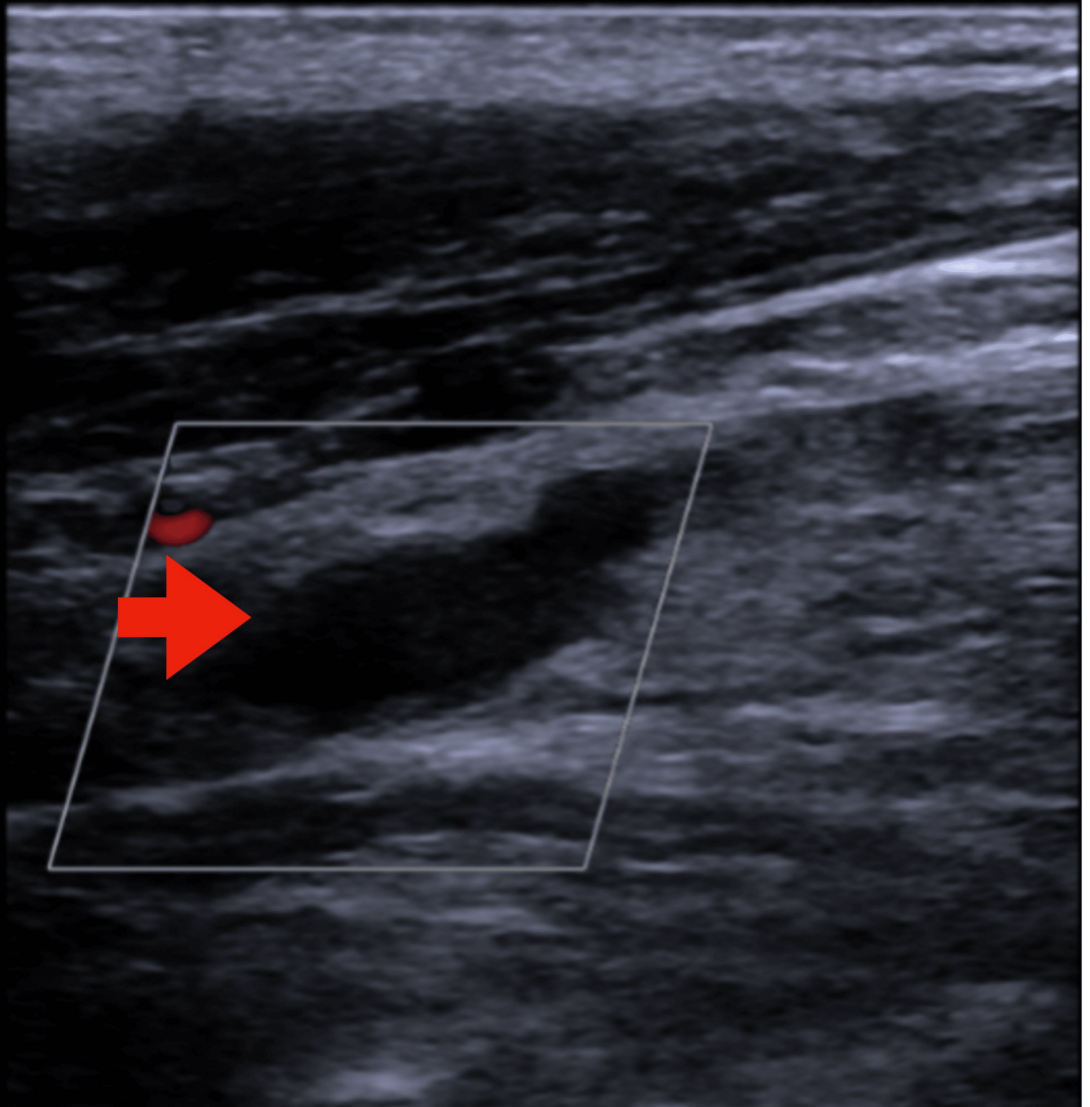
Venogram showing the right subclavian occlusion.

**Figure 2:**
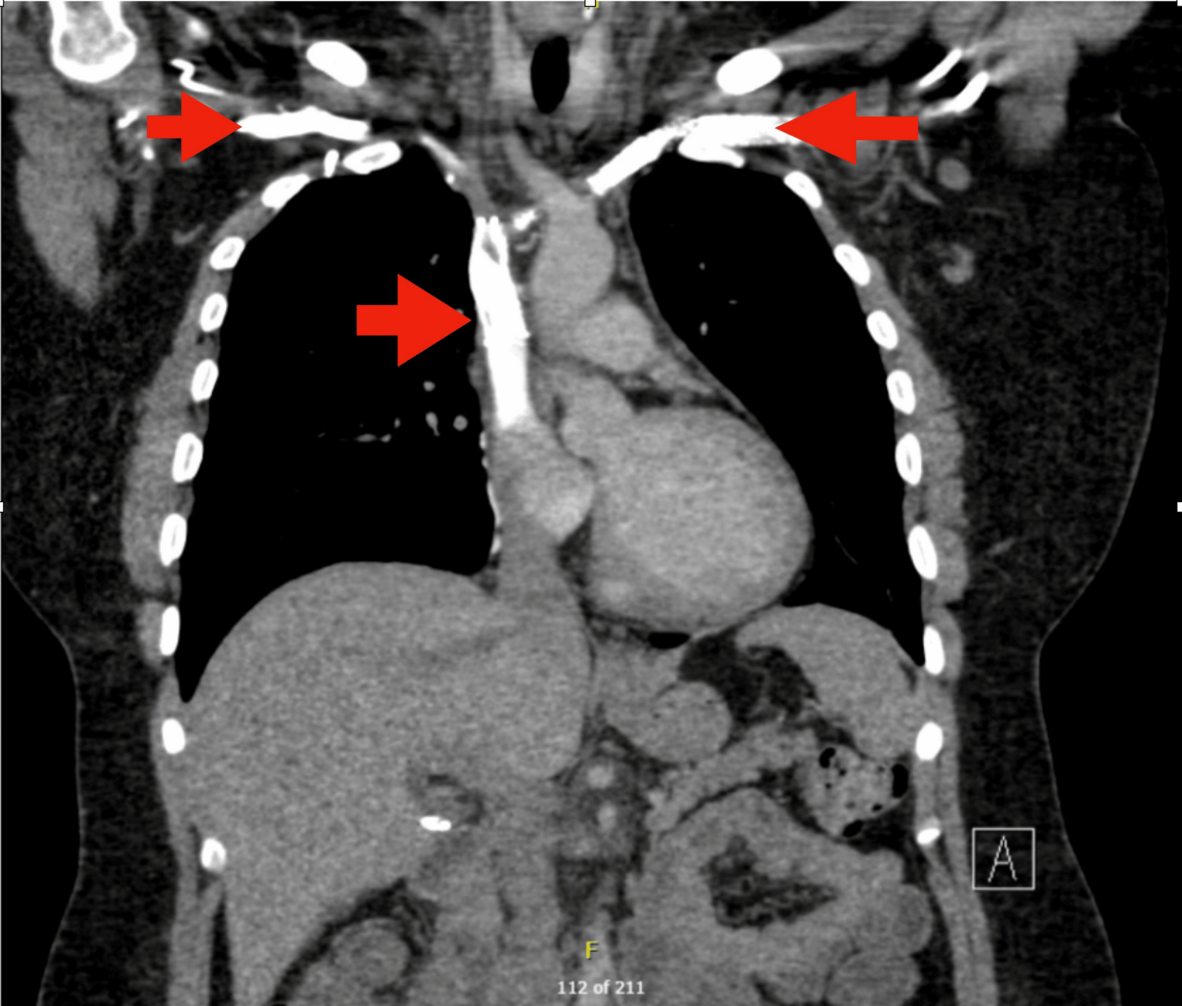
Mild narrowing of the left subclavian venous stent between the left first rib and clavicle. Filling defects in the stented left brachiocephalic vein and SVC, suspicious for in-stent thrombi. Chronic occlusion/severe narrowing of the right brachiocephalic and right subclavian veins. New solid nodular opacity in the right upper lobe, favored infectious in the acute setting.

**Figure 3:**
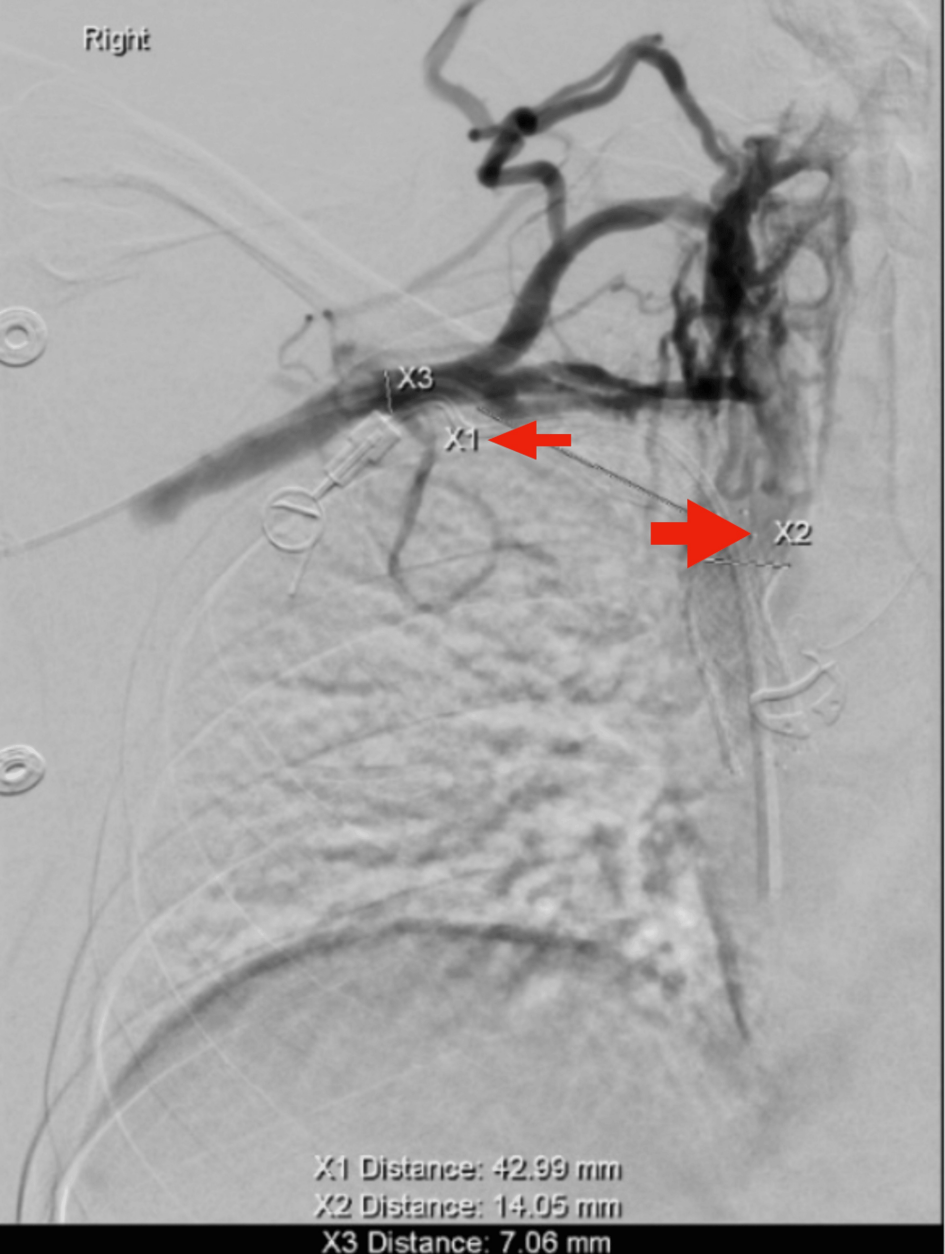
Right upper extremity venography performed in November 2022 demonstrating complete occlusion of the right subclavian vein extending from the thoracic inlet to its junction with the superior vena cava. No contrast opacification is seen through the subclavian vein, and extensive venous collaterals are noted along the chest wall and neck, indicating chronic obstruction. Measurement markers (X1–X3) were used intraoperatively to assess occlusion length (≈43 mm) and vessel diameter. These findings are consistent with right-sided venous thoracic outlet syndrome.

On June 25, bilateral venography (Figure 1) with pressure measurements demonstrated patent left-sided stents and complete occlusion of the right subclavian vein from the clavicle to its junction with the SVC, consistent with bilateral TOS. Venous pressures were normal as they read: 10 mmHg in the left brachial, axillary, subclavian, brachiocephalic veins, and SVC and pressure of 7 mmHg in the right atrium. Associated CT angiography findings showed mild narrowing of the left subclavian venous stent between the left first rib and clavicle, filling defects in the stented left brachiocephalic vein and SVC suspicious for in-stent thrombi, and chronic occlusion/severe narrowing of the right brachiocephalic and right subclavian veins (Figure 2). These findings were concordant with a prior right upper extremity venogram performed in November 2022, which had similarly demonstrated complete occlusion of the right subclavian vein with extensive venous collaterals, consistent with chronic right-sided venous TOS (Figure 3). The patient was given heparin as an anticoagulant. Thoracic surgery scheduled a left first rib resection for July 3, 2025, with perioperative Coumadin held. Surgery was later canceled as per the surgeon's request do to the high-risk nature of the patients' extensive bilateral chronic venous occlusion, in-stent thrombosis, and APS-related hypercoagulability.

During hospitalization, she experienced six episodes of green-brown diarrhea over 24 hours, attributed to a prolonged nil per os status. There was no abdominal colic or evidence of infectious colitis. Symptoms were managed conservatively with acetaminophen and tramadol as needed.

## Discussion

This case highlights the multifactorial approach and challenges encountered in a case that concurrently presents with APS, SVCS, and venous thoracic outlet syndrome (vTOS). Both vTOS and SVCS are individually uncommon disorders of venous obstruction [1-4]. When compounded by APS - a prothrombotic autoimmune condition characterized by pathogenic antiphospholipid antibodies - the risk of both initial and recurrent thrombosis becomes markedly increased, even under aggressive anticoagulation.

APS-related thrombosis is driven by endothelial activation, complement-mediated injury, and upregulation of tissue factor expression, all of which sustain a chronic hypercoagulable milieu. In most patients, long-term anticoagulation with vitamin K antagonists, such as warfarin, remains the mainstay of therapy [5]. However, in this case, even the traditional high-intensity warfarin therapy with supratherapeutic INR levels failed to prevent recurrent thromboses, underscoring the limitations of conventional therapy and highlighting a subset of “anticoagulation-resistant APS” [6-8]. Such resistance may be resultant from alternative prothrombotic mechanisms independent of vitamin K pathways, including persistent endothelial dysfunction, complement activation, or secondary triggers such as systemic inflammation and infection.

The coexistence of hereditary angioedema and mast cell activation syndrome further complicated management. Both conditions contribute to recurrent gastrointestinal dysmotility, abdominal pain, and protein-losing enteropathy, leading to malabsorption and nutritional compromise. These chronic nutritional deficits necessitated dependence on central venous access for TPN, which in turn introduced additional iatrogenic risks of catheter-related bloodstream infection and septic thrombophlebitis [9-11]. The need for management during such episodes puts the patient at risk of exacerbation of venous inflammation, which can result in further vascular injury, inflammation, and further propagation of thrombotic events.

The nature of such a complex case illustrated the need for a multidisciplinary coordination for the evaluation and treatment of mechanical decompression, infectious disease control, gastroenterology for nutrition optimization, and immunological evaluation. Moreover, the case reinforces the importance of individualized treatment strategies, such as exploring non-vitamin K anticoagulants, complement inhibitors, or targeted biologic therapies.

Finally, the patient’s presentation aligns with the broader understanding that systemic autoimmune and prothrombotic disorders have a higher prevalence and severity in females, likely influenced by hormonal, genetic, and epigenetic factors that modulate immune tolerance and vascular reactivity [12]. It serves as a reminder that success in such patients depends less on adherence to a single therapeutic algorithm and more on a dynamic, patient-specific approach emphasizing early detection, multidisciplinary management, and a high index of suspicion for recurrent or atypical thrombosis.

## Conclusions

This case highlights the complex relationship between mechanical vascular compression and systemic hypercoagulability in a patient with treatment-resistant APS complicated by concurrent TOS and SVCS. Despite high-intensity warfarin therapy, recurrent thrombotic events occurred, underscoring the limitations of standard anticoagulation in high-risk APS patients. The coexistence of hereditary angioedema and mast cell activation further complicates management by contributing to gastrointestinal dysmotility, nutritional dependence, and increased infection risk. Optimal care requires a multidisciplinary approach integrating hematology, vascular surgery, immunology, and infectious disease expertise. This case underscores the urgent need for individualized treatment strategies and highlights gaps in current guidelines for managing patients with overlapping autoimmune, vascular, and rare genetic disorders. Further research is essential to develop tailored anticoagulation protocols and comprehensive multidisciplinary care models for these challenging cases.
